# Knowledge-based immune-therapeutic advances for transplantation and cancer

**DOI:** 10.1093/immadv/ltaf014

**Published:** 2025-03-28

**Authors:** Elizabeth Simpson

**Affiliations:** Emeritus Professor of Transplantation Biology, Division of Immunology & Inflammation, Department of Medicine, Imperial College, London, United Kingdom

**Keywords:** retrospective, transplantation immunology, cancer immunology

## Abstract

Increased knowledge about immune responses to what is perceived as ‘foreign’ or ‘non-self’ has revealed extraordinary levels of complexity. Research uncovering these has the potential to optimise immunotherapy. Remarkable progress has been made in transplantation leading to much less acute rejection and a very significant increase in long-term survival. These and other beneficial results have been achieved by stepwise advances, using combinations of less toxic chemotherapies with biological immunosuppressive agents alongside innovatory surgical skills. However, rejection is the default pathway for entities deemed ‘foreign’, including mistaken suspects like autoantigens. For tumour immunotherapy the identification of *bona fide* tumour antigens recognised by B and/or T cell receptors has mushroomed but has yet to be optimised to take into account mutations leading to tumour evasion. We are now at the beginning of a road that looks more hopeful by being informed of the complexities. Basic and translational research between the late 1960s and now have yielded an extraordinarily rich harvest of new knowledge. More is to come, further insight is needed and new paradigms explored.

In 1969, as a veterinary graduate with postgraduate training in pathology and an MRC project grant to explore tumour immunology, I joined the National Institute for Medical Research (NIMR) at Mill Hill. There the Director, Peter Medawar, encouraged the staff—biochemists, microbiologists, parasitologists and immunologists, to work imaginatively and collaboratively at cutting edge questions. It was a very exciting and supportive environment, and one in which as a neophyte I had a great deal to learn.

In this review I’ve selected the key papers that influenced my starting point and approach, which was to use ‘weak’ minor histocompatibility antigens as proxies for tumour antigens. It seemed to me that exploring underlying basic mechanisms was essential both for tumour immunotherapy and control of graft rejection.

The references cited include some chosen to illustrate the narrative alongside those I view as landmarks with respect to tolerance [4, 5], MHC antigens [7], immune response genes [14], MHC restriction [15], monoclonal antibodies [17], immunoglobulin and T cell receptor (TCR) genes [18, 20], peptide targets of viruses other antigens [22, 23, 25, 26, 30, 33], and as an example of molecules in activation pathways [36]. Landmark references are asterisked throughout the text.

At the time I started in the late 1960s, if considered at all, T cells tended to be regarded as epiphenomena. However, during my postgraduate training in pathology I was struck by the similarity between cellular infiltrates in granulomas (eg TB lesions) with those around tumours in biopsies from human or veterinary patients with good prognoses. Reading Medawar’s papers on the histology of skin graft rejection [1, 2] I was convinced by the argument that ‘cell mediated immunity’ was different from the antibody response.

This view had been confirmed by transfer of skin graft rejection with cells, rather than serum—a key experiment by Mitchison [3] when a PhD student of Medawar. That paper was published in the same year, 1953, as another key publication from the Medawar lab, by Billingham, Brent and Medawar (4*), on the experimental induction of donor specific transplantation tolerance in mice, later reproduced in other species (5*), thus opening a pathway to therapeutic approaches for clinical transplantation. Medawar’s subsequent research on the use of antilymphocyte serum for immunosuppression showed prolongation skin graft survival in mice, Lance, Levey, Medawar and Ruszkiewicz [6], but not specificity. Nor did the use of cytotoxic drugs in clinical transplantation, first developed to treat cancer patients, address this issue, as they too induced general immunosuppression.

Progress was also hampered by not knowing the identity of target antigen(s) of graft rejection. In the 1940s George Snell (7*) had taken a genetic approach by using inbred mouse strains. He distinguished ‘strong’ histocompatibility antigens evoking rapid graft rejection from a category of ‘weak’, or minor histocompatibility antigens inducing slower rejection. His results using inbred mouse strains showed that histocompatibility (H) genes were located in many independently segregating genetic loci which he set about mapping. Most were autosomal, in ‘linkage groups’, on what were then unnumbered chromosomes, but he also named a male-specific transplant antigen, HY, as a locus on the Y chromosome, thus present only in males. The other H loci were numbered in the order in which they were identified in Snell’s backcross experiments. The strongest, H2, elicited the most rapid skin graft rejection in all combinations so was designated the ‘major histocompatibility’ locus, later ‘complex’ (MHC).

Use of genetics was also key for identifying the human MHC equivalent, HLA. During a series of international workshops in the 1960s the efforts of a number of clinically motivated teams in France, Holland, Italy and the USA, working with sera from blood samples of multiparous women, the HLA ‘locus’ on human chromosome 6 was determined. It appeared to be a complex of closely linked multi-allelic genes, initially considered as components of at least 2 loci, HLA.A and HLA.B with HLA.C later added as a third serologically encoded locus of MHC class I molecules Bodmer [8]. Parallel research using a combination of serology and proliferative responses in mixed lymphocyte cultures revealed further multiallelic genes encoding MHC class II molecules, their loci in due course named HLA.D. Schendel and Bach [9].

These key discoveries for exploring how the immune system works were recognised by Nobel prizes to Medawar in 1960 for his work on tolerance to transplants and in 1980, to Snell and Dausset for theirs on the identification of MHC encoded targets of graft rejection in mice and humans respectively. In due course MHC genes were found to control all immune responses in which T cells played a role.

This area of discovery was expanding when I started research in 1969 at the National Institute of Medical Research (NIMR) in Mill Hill, London, and I continued working on the T cell side of it through the 1970s and beyond, at NIMR and in the USA at NIH, Stanford and the Jackson Laboratories in Maine. Advances in immunology were co-dependent on those in genetics, and thence molecular biology. Neophytes needed to have broad imaginations.

Medawar’s National Institute of Medical Research (NIMR) was a hot house of cutting-edge ideas about immunity. There I joined other scientists interested in the function of T cells, working towards developing *in vitro* methods to match *in vivo* observations. A powerful starting point was the delineation of T cells, initially in relation to distinguishing them from antibody producing B lymphocytes and then extending the analysis to identify functionally distinct subpopulations of T cells, for which short and long-term *in vitro* culture methods were needed. Having begun along this track in NIMR, I took up the offer of a research fellowship in the USA at National Institutes of Health (NIH) to work in the lab of John Wunderlich [10], and to collaborate with other NIH immunologists. These included Charlie Janeway and Harvey Cantor, and later in Stanford with Len Herzenburg [10–13] using his newly developed fluorescence activated cell sorter (FACS) to test the phenotype and function of sorter-separated T cell subpopulations before and after *in vitro* culture.

My focus was on cytotoxic T cells, and one of my findings at NIH was that lymphocytes from mice previously immunised with cells from MHC H2 discrepant strains *in vivo* subsequently made enhanced *in vitro* responses, with increased levels of proliferation and generation of cytotoxic T cells [10]. This suggested to me an approach for generating *in vitro* T cell responses to minor (weak, non-H2) histocompatibility antigens that did not, like H2 disparities, elicit primary cytotoxic responses *in vitro*.

When I returned to London in 1973 as a group leader in the MRC’s new Clinical Research Centre (CRC) I decided to work on identifying the male specific transplantation antigen, HY, because it was genetically well defined and located on the smallest mouse chromosome, seeming to offer a tractable target for molecular identification. We had a flying start, developing HY specific cytotoxic T cells from responder strains like C57Bl/6, but not some other laboratory strains like CBA. This responder/non-responder phenotype to HY had been found *in vivo* by Donald Bailey, at the Jackson Lab in Maine, to be linked to MHC genotype, with females of all H2^b^ strains tested able to reject skin grafts from males of the same strain, a response not found in inbred strains of other H2 haplotypes. I knew that Hugh McDevitt (14*) had discovered evidence for genetic control of antibody responses to haptens, so comparable findings for T-cell responses opened the prospect of exploring this with HY using *in vitro* approaches.

Peter Doherty and Rolf Zinkernagel’s findings on MHC restriction of cytotoxic T-cell responses to viruses, published as a letter to Nature in 1974 (15*), were presented that year at an International Transplantation Society workshop that I chaired, allowing me to glimpse the implications for T-cell responses to a wider range of targets. I returned to my lab after that workshop to discover that our experiments exploring the target cell specificity of male specific cytotoxic T cells fitted into this pattern—an exciting finding that we published in JEM in 1975 [16].

Doherty and Zinkernagel’s MHC restriction hypothesis, that T cells used receptors that recognised two moieties, one determined by self MHC, the other by a non-self-component specified by a virus or other antigen, caused a great deal of discussion and argument. The implication that the exquisitely specific immunoglobulin receptors of B cells were not used by T cells involved a paradigm shift. This happened as studies of immunoglobulin were moved to another plane by Milstein and Kohler’s landmark discovery in 1975 of how to make monoclonal antibodies (17*), the foundational discovery for developing monoclonal antibodies for clinical use in diagnostics and therapy. This was enthusiastically pursued on both sides of the Atlantic and worldwide. Seeking patents on monoclonal antibodies was contentious but countered by arguments of the need for significant further funding to address questions about safety and efficacy for clinical use.

Crucially, basic research continued alongside clinical application in order to obtain deeper understanding of mechanisms and open up new areas for exploration. Tonegawa’s results identifying somatic generation of antibody diversity (18*), was shown as due to recombination of different light and heavy chain immunoglobulin gene segments plus junctional diversity.

As it became clear that the specificity of T cells was as variable and exquisitely discriminatory as that of B cells, the hunt for T cell receptor homologues of immunoglobulin light and heavy chains were sought—and eventually found (eg, (19,20*). Expression of these defined subpopulations with alpha, beta or gamma T cell receptors and a range of functions according to T cell phenotypes and characterised by the cell surface expression of CD4 and CD8 molecules induced during thymic differentiation, as well as differential expression of cytokines and cytokine/chemokine receptors.

Within the framework of research and translation for controlling rejection of tissue grafts Waldman [21] explored the use of monoclonal antibodies (MAb) specific for T cell specific molecules such as CD4, CD8 and CD3, first in mouse skin grafting experiments, followed by the development of humanised MAb for inclusion in treatment of bone marrow and kidney graft recipients and as well as for patients with certain autoimmune diseases like multiple sclerosis. In many cases therapeutic MAbs allowed reduction in the dosage of toxic immunosuppressive drugs.

For control of transplant rejection and autoimmune responses, dampening of immunity is required, whereas for tumour immunotherapy amplifying the response is needed. These are two sides of the same coin and the results of research in each is relevant to the other for clinical application.

The use of monoclonal reagents was extended to tumour immunotherapy, first using MAb against cell surface molecules, such as CD19 expressed by B cells and their tumours or the clonal immunoglobulin on myelomas. But before continuing with a further account of this approach, it is necessary to consider research on monoclonal reagents derived from antigen specific T cells, requiring isolation of T cells of appropriate specificity for identifying the non-MHC component of their target antigen.

Alain Townsend’s research to identify the flu peptide recognised by well characterised cytotoxic cells (22*) was the last piece in solving the puzzle about the two components recognised by T cell receptors, enabling interpretation by Pam Bjorkmann in Don Wiley’s lab of the ‘dual recognition’ target of T cells, graphically illustrated with the crystal structural image of an HLA class I molecule folded to create the groove enfolding the peptide component (23*). This was extended to T cells recognising their somewhat longer target peptides in the context of MHC class II molecules. Thereafter publication of the crystal structures of a series of both MHC class I and II restricted T cells gave further insight into the structure and function of TCR molecules for use in immunotherapy clinically. But there was and is still further enabling research needed.

What was still lacking were ways to molecularly identify from large libraries the components of non-viral MHC restricted antigens recognised by T cells, such as tumour antigens and autoantigens. By the beginning of the 1980s, following Thierry Boon’s isolation of T cell clones specific for tumour antigens and our development of HY specific T cell clones restricted by a range of MHC class I and II molecules, it became clear that their use for identification of target antigens required parallel new developments in cell and molecular biology. Although results of transfecting candidate DNA fragments into indicator cells could be screened with antibodies, Mellor & Flavell 1982 [24], the use of T cells with their cell-bound receptors to identify and select rare positive transfectants after transfection of DNA or cDNA libraries, posed problems for exploring the potential of such monoclonal T cells and their receptors for clinical use. The *in vitro* expansion of antigen specific effector clones for therapeutic transfer *in vivo* required this development as well as for the molecular cloning of their T cell receptors (TCRs) for use as templates for therapeutics.

Thierry Boon (25*) in Brussels and Nilabh Shastri [26] in the USA, focusing on tumour antigens and minor H antigens respectively, sought ways of using T cells to detect rare positive transfectants. Diane Scott [27, 28] in our lab worked to develop methods for screening candidate genes isolated from chromosomally defined locations. With our Jackson Lab colleagues, Derry Roopenian and Eva Eicher [29], we used a genetic approach based on immunoselection and variable X chromosome inactivation.

Genetic mapping of the Y chromosome with Anne McLaren (30*) allowed us to identify a deletion mutant that separated our HY genes from the testis determining gene Sry as the first step for cloning and testing the function of each of these genes *in vivo* [30, 31]. Homologues of the mouse HY genes were found in humans [32–34] with potential for therapeutic use in treatment of leukaemia [35].

Subsequent identification and isolation of tumour specific T cell receptor molecules has allowed them to be incorporated in CAR T cells or transduced into recipient effector cells. Additional novel approaches have involved targeting molecules in key activation and/or differentiation pathways and include powerful check-point inhibitors [36]. As these pioneering tumour immunotherapy approaches have proceeded through clinical trial, the number of curative responses is often limited to subsets of patients, probably due to heterogeneity of the patients and/or the disease. Combination therapies are edging forward into this territory, much as they have done in transplantation for which the holy grail of antigen specific to tolerance has proved elusive.

Increased knowledge about immune responses to what is perceived as ‘foreign’ or ‘non-self’ has revealed extraordinary levels of complexity. Research uncovering these has the potential to optimise immunotherapy. Remarkable progress has been made in transplantation resulting in much less acute rejection and a very significant increase in long-term survival. These and other beneficial results have been achieved by stepwise advances, using combinations of less toxic chemotherapies with biological immunosuppressive agents alongside innovatory surgical skills. However, rejection is the default pathway for entities deemed ‘foreign’, including mistaken suspects like autoantigens. For tumour immunotherapy the identification of *bona fide* tumour antigens recognised by B and/or T cell receptors has mushroomed but has yet to be optimised to take into account mutations leading to tumour evasion. We are now at the beginning of a road that looks more hopeful by being informed of the complexities. Basic and translational research between the late 1960s and now have yielded an extraordinarily rich harvest of new knowledge. More is to come, further insight is needed and new paradigms explored.

More detailed historical references can be found in three books published between 1975 and 2013 [37–39]. The first chapters of Leslie Brent’s ‘History of Transplantation Immunology’ [37] provide links to earlier research relevant to transplantation and the cellular and molecular immunology that followed. Jan Klein’s ‘Biology of the Mouse H2 complex’ [38] is a passionate, scholarly survey focused on the evolutionary genetics of wild mice and the laboratory strains developed from them. Daniel Davis in his opening chapters provides a clear-sighted view of interaction between scientists working on histocompatibility genes as advances at the cellular and ultrastructural advances illuminate function.

## Data Availability

Not applicable.

## References

[1] Medawar PB. The behaviour and fate of skin autografts and skin homografts in rabbits: a report to the War Wounds Committee of the Medical Research Council. J Anat 1944; 78:176–99. 17104960 PMC1272490

[2] Medawar PB. A second study of the behaviour and fate of skin homografts in rabbits: a report to the War Wounds Committee of the Medical Research Council. J Anat1945; 79:157–176.4.21004505

[3] Mitchison NA. Passive transfer of transplantation immunity. Nature 1953; 171:267–8. https://doi.org/10.1038/171267b0 13036857

[4] *Billingham R , Brent L, Medawar PB. ‘Actively acquired tolerance’ of foreign cells. Nature 1953; 172:603–606. 13099277 10.1038/172603a0

[5] *Billingham B ; Medawar. ‘Quantitative studies on tissue transplantation immunity. III. Actively acquired tolerance. Phil.Trans. R. Soc. B 1956; 239:357–414.

[6] Lance EM , Levey RH, Medawar PB et al Tolerance of rat skin grafts in adult mice. Proc Natl Acad Sci U S A 1969; 64:1356–61. https://doi.org/10.1073/pnas.64.4.1356 4393916 PMC223292

[7] *Snell GD. Methods for the study of histocompatibility genes. J.Genet 1948; 49:87–108. 18893744 10.1007/BF02986826

[8] Bodmer W , A historical perspective on HLA. Immunother Adv 2023; 3:1. 10.1093/immadv/ltad014 PMC10449411 37636243

[9] Schendel DJ , Alter BJ, Bach FH. The involvement of LD- and SD-regions differences in MLC and CML: a three-cell experiment. Transplant Proc 1973; 5:1651–5. 4272823

[10] Simpson E , O’Hopp S, Wunderlich J. Life span of cytotoxic activity and memory activity following allogeneic skin grafting in the mouse. Transplantation 1974; 18:374–7. https://doi.org/10.1097/00007890-197410000-00014 4607454

[11] Janeway CA , Sharrow SO, Simpson E. T cell populations with different functions. Nature 1975; 253:544–6. https://doi.org/10.1038/253544a0 1078881

[12] Cantor H , Simpson E. Regulation of the immune response by subclasses of T lymphocytes. I. Interactions between peripheral lymphoid tissues of mice. Eur J Immunol 1975; 5:330–6. 1086234 10.1002/eji.1830050508

[13] Cantor H , Simpson E, Sato V et al Characterisation of subpopulations of T lymphocytes, I. Identification of T cell subpopulations according to amount of cell-bound Thy 1.2 (Theta). Cell Immunol 1975; 25:280. 10.1016/0008-8749(75)90174-4 1088903

[14] *McDevitt HO , Sela M. Genetic control of the antibody response: demonstration of determinant-specific differences in response to synthetic polypeptide antigens in two strains of inbred mice. J Exp Med 1965; 122:517–31. 5839284 10.1084/jem.122.3.517 PMC2138070

[15] *Zinkernagel RM , Doherty PC. Restriction of in vitro T cell-mediated cytotoxicity in lymphocytic choriomeningitis within a syngeneic or semiallogeneic system. Nature 1974; 248:701–2. 4133807 10.1038/248701a0

[16] Gordon R , Simpson E, Samelson L. *In vitro* cell-mediated immune responses to the male specific (H-Y) antigen in mice. J Exp Med 1975; 142:1108–20. 1081575 10.1084/jem.142.5.1108 PMC2189978

[17] *Kohler G , Milstein C. Continuous cultures of fused cells secreting antibody of predefined specificity. Nature 1975; 256:495–497. 1172191 10.1038/256495a0

[18] *Tonegawa S. Reiteration frequency of immunoglobulin light chain genes: further evidence for somatic generation of antibody diversity. Proc Natl Acad Sci USA 1976; 73:203–207. 813222 10.1073/pnas.73.1.203 PMC335869

[19] Raulet DH , Garman RD, Saito H et al Developmental regulation of T-cell receptor gene expression. Nature 1985; 314:103–7. https://doi.org/10.1038/314103a0 2983227

[20] *Davis MM , Chien YH, Gascoigne NR et al A murine T cell receptor gene complex: isolation, structure and rearrangement. Immunol Rev 1984; 81:235–58. 6096259 10.1111/j.1600-065x.1984.tb01113.x

[21] Qin SX , Wise M, Cobbold SP et al Induction of tolerance in peripheral T cells with mono-clonal antibodies. Eur J Immunol 1990; 20:2737–45. https://doi.org/10.1002/eji.1830201231 1702726

[22] *Townsend AR , Gotch FM, Davey J. Cytotoxic T cells recognize fragments of the influenza nucleoprotein. Cell 1985; 42:467. 10.1016/0092-8674(85)90103-5 2411422

[23] *Bjorkman PJ , Saper MA, Samraoui B et al The foreign antigen binding site and T cell recognition regions of class I histocompatibility antigens. Nature 1987; 329:518. 10.1038/329512a0 2443855

[24] Mellor AL , Golden L, Weiss E et al Expression of the murine H-2K^b^ histocompatibility antigen in cells transformed with cloned H-2 genes. Nature 1982; 298:1982. 10.1038/298529a0 6285197

[25] *De Plaen E , Lurquin C, Van Pel A et al Immunogenic (tum-) variants of mouse tumor P815: cloning of the gene of tum- antigen P91A and identification of the tum- mutation. Proc Natl Acad Sci U S A 1988; 85:2274–8. 3127830 10.1073/pnas.85.7.2274 PMC279973

[26] Sahara H , Shastri N. Second class minors: molecular identification of the autosomal H46 histocompatibility locus as a peptide presented by major histocompatibility complex class II molecules. J Exp Med 2003; 197:375–85. https://doi.org/10.1084/jem.20021961 12566421 PMC2193838

[27] Scott D , McLaren A, Dyson J et al Variable spread of X inactivation affecting the expression of different epitopes of the Hya gene product in mouse B cell clones. Immunogenetics 1991; 33:54–61. https://doi.org/10.1007/BF00211696 1995476

[28] Scott D , Dyson J, Simpson E. A new approach to the cloning of genes encoding T cell epitopes. Immunogenetics 1992; 36:86–94. 1377172 10.1007/BF00215284

[29] King TR , Christianson GJ, Mitchell MJ et al Deletion mapping using immunoselection for H-Y further resolves the Sxr region of the mouse Y chromosome and reveals complexity at the Hya locus. Genomics 1994; 24:159–68. 7896271 10.1006/geno.1994.1593

[30] McLaren A , Simpson E, Tomonari K et al Male sexual differentiation in mice lacking H-Y antigen. Nature 1984; 312:552–5. 6542174 10.1038/312552a0

[31] Koopman P , Gubbay J, Vivian N, Goodfellow P, Lovell-Badge R. Male development of chromosomally female mice transgenic for *Sry. Nature.* 1991; 351:117-21. https://doi.org/10.1038/351117a0 2030730

[32] Simpson E , Chandler P, Goulmy E et al Separation of the genetic loci for the H-Y antigen and testis determination on the human Y chromosome. Nature 1987; 326:876–8. https://doi.org/10.1038/326876a0 3494951

[33] *Scott DM , Ehrmann IE, Ellis PS et al Identification of a mouse male-specific transplantation antigen, H-Y. Nature 1995; 376:695–8. 7544442 10.1038/376695a0

[34] Greenfield A , Scott D, Pennisi D et al An H-Y D^b^ epitope is encoded by a novel mouse Y chromosome gene. Nature Genet 1996; 14:474–8. 8944031 10.1038/ng1296-474

[35] Warren EH , Gavin MA, Simpson E et al The human Uty gene encodes a novel HLA-B8-restricted HY antigen. J Immunol 2000; 164:2807–14. https://doi.org/10.4049/jimmunol.164.5.2807 10679124

[36] *Padmanee S , Allison J et al Immune checkpoint therapy-current perspectives and future directions. Cell 2023; 186:1652–166. 37059068 10.1016/j.cell.2023.03.006

[37] Brent L. A History of Transplantation Immunology. Cambridge, MA: Academic Press, 1996.

[38] Klein J. Biology of the Mouse Histocompatibility-2 Complex. Principles of Immunogenetics Applied to a Single System. New York: Springer-Verlag, 1975.

[39] Davis D. The Histocompatibility Gene. Oxford, UK: Oxford University Press, 2013.

